# Reacquisition of New Meristematic Sites Determines the Development of a New Organ, the Cecidomyiidae Gall on *Copaifera langsdorffii* Desf. (Fabaceae)

**DOI:** 10.3389/fpls.2017.01622

**Published:** 2017-09-27

**Authors:** Renê G. S. Carneiro, Rosy M. S. Isaias, Ana S. F. P. Moreira, Denis C. Oliveira

**Affiliations:** ^1^Laboratório de Anatomia Vegetal, Instituto de Ciências Biológicas, Universidade Federal de Goiás, Goiânia, Brazil; ^2^Laboratório de Anatomia Vegetal, Instituto de Ciências Biológicas, Universidade Federal de Minas Gerais, Belo Horizonte, Brazil; ^3^Laboratório de Fisiologia Vegetal, Instituto de Biologia, Universidade Federal de Uberlândia, Uberlândia, Brazil; ^4^Laboratório de Anatomia e Desenvolvimento Vegetal e Interações, Instituto de Biologia, Universidade Federal de Uberlândia, Uberlândia, Brazil

**Keywords:** cell redifferentiation, gall shape, hystochemistry, photosynthesis

## Abstract

The development of gall shapes has been attributed to the feeding behavior of the galling insects and how the host tissues react to galling stimuli, which ultimately culminate in a variable set of structural responses. A superhost of galling herbivores, *Copaifera langsdorffii*, hosts a bizarre “horn-shaped” leaflet gall morphotype induced by an unidentified species of Diptera: Cecidomyiidae. By studying the development of this gall morphotype under the anatomical and physiological perspectives, we demonstrate the symptoms of the Cecidomyiidae manipulation over plant tissues, toward the cell redifferentiation and tissue neoformation. The most prominent feature of this gall is the shifting in shape from growth and development phase toward maturation, which imply in metabolites accumulation detected by histochemical tests in meristem-like group of cells within gall structure. We hypothesize that the development of complex galls, such as the horn-shaped demands the reacquisition of cell meristematic competence. Also, as mature galls are green, their photosynthetic activity should be sufficient for their oxygenation, thus compensating the low gas diffusion through the compacted gall parenchyma. We currently conclude that the galling Cecidomyiidae triggers the establishment of new sites of meristematic tissues, which are ultimately responsible for shifting from the young conical to the mature horn-shaped gall morphotype. Accordingly, the conservative photosynthesis activity in gall site maintains tissue homeostasis by avoiding hypoxia and hipercarbia in the highly compacted gall tissues.

## Introduction

For millions of years, insects and plants have developed strategies of attack and counter attack that culminate in the establishment of unique ecological relationships between living organisms (Howe and Jander, 2008). One of the most intriguing of such interactions leads to the development of galls, with several morphotypes that generally result from species-specific relationships (Stone and Schönrogge, 2003; Isaias et al., 2013). Therefore, specialized insects are capable of manipulating plant tissues and metabolism to induce a neo-formed plant organ, the gall (Mani, 1964; Shorthouse et al., 2005; Giron et al., 2016, Oliveira et al., 2016). Additionally, a cascade of subcellular events related to the increased oxidative stress and the mechanisms of stress control, cell signaling, division, and elongation should play significant roles in gall development and determination of gall shape (Mittapalli et al., 2007; Oliveira and Isaias, 2010; Oliveira et al., 2010, 2016; Bedetti et al., 2014; Isaias et al., 2015).

In the past two decades, there has been an effort to elucidate the cellular mechanisms of determination of gall shapes commonly observed among Neotropical galls (*cf*. Isaias et al., 2013). Even simple-shaped galls such as the globoid, lenticular, and fusiform seem to develop by a variety of somewhat divergent cellular dynamics (Oliveira et al., 2010, 2016; Carneiro et al., 2014, 2015; Isaias et al., 2014a; Suzuki et al., 2015), but complex bizarre-shaped gall morphotypes demand much deeper alterations in the fates of plant host cells and tissues.

The best way to reprogram cell cycles seems to be by turning cells back to the meristematic condition, which has not been observed in anatomical studies concerning the globoid, fusiform and lenticular galls (Oliveira et al., 2010; Carneiro et al., 2013; Isaias et al., 2013, 2014b; Vecchi et al., 2013). However, in the bivalve-shaped gall induced by *Euphalerus ostreoides* on *Lonchocarpus muehlbergianus* (Isaias et al., 2011) groups of redifferentiated cells are responsible for the development of two valves that protrude from the leaf lamina to form the larval chamber. Herein, we propose that alterations on the degree of cell redifferentiation in galls depend on refined stimulation by galling insects, and the reacquisition of meristematic condition, i.e., dedifferentiation (*sensu* Buvat, 1989). We hypothesize that a profound cell redifferentitation enable some galls to assume complex forms, sometimes very distinct from the most common gall morphotypes observed in nature (Isaias et al., 2013, 2014b), such as the bizarre horn-shaped galls induced on leaflets of *Copaifera langsdorffii*.

Leaves are the most common oviposition sites for galling herbivores (Mani, 1964; Isaias et al., 2013; Fernandes and Santos, 2014), which alter the structure and function of the tissues, sometimes, in detriment of their photosynthetic capacity. For instance, some galls may photosynthesize as much as their host leaves, as reported for the galls induced by *Pseudophacopteron aspidospermii* (Malenovsky et al., 2015) on *Aspidosperma australe*, and a Cecidomyiidae on *Aspidosperma spruceanum* (Oliveira et al., 2011), or may have depleted photosynthetic apparatus (Larson, 1998; Kar et al., 2013). The horn-shaped galls of *C. langsdorffii* seem to belong to the group of photosynthesis deficient galls, for their levels of total chlorophyll are about 27-fold lower than the non-galled leaflets, while the maximum electron transport rates decrease about seven times (Castro et al., 2012). However, even at low rates, the photosynthesis in green galls may provide oxygen to avoid hypoxia, and consume carbon dioxide to avoid hypercarbia, thus helping to maintain the stability of the organ (Pincebourde and Casas, 2016; Oliveira et al., 2017).

Current study focuses on the “horn-shaped” leaflet gall morphotype induced by a Cecidomyiidae (Diptera) on the superhost of galling herbivores, *C. langsdorffii* (Fabaceae). Field observations revealed a strong alteration in shape from young to mature galls (Isaias et al., 2014a), besides great increase in size. We expect that, by studying the development of such gall morphotype under the anatomical and physiological perspectives, we can elucidate key steps involving the transition from the laminar structure of non-galled leaves to the conical shape of young galls, and toward the mature horn-shaped galls. In addition, we expect that both outer and inner gall tissue compartments (Bragança et al., 2017) should maintain some level of photosynthesis, which is important to the metabolic stability of the gall.

## Materials and Methods

### Studied Area and Plant Material

Samples of the galls and non-galled leaflets were collected in a population of *C. langsdorffii* Desf. (Fabaceae) growing on ferruginous rocky outcrops (*canga*) at Retiro das Pedras (20°05“35”S, 43°59“01”W), Serra da Calçada, municipality of Brumadinho, southeastern Brazil. The population (15 plant individuals bearing hundreds of galls, each) was monitored and sampled monthly (*n* ≥ 5 galls and non-galled leaves) from January 2007 to March 2008, to define the different developmental stages of the horn-shaped gall.

### Developmental Anatomy and Determination of Gall Stages

For structural studies, galls in successive stages of development (*n* = 5 *per* stage) were separated according to their sizes and morphological features. Induction stage was defined by a swelling on the leaf lamina (1.0 ± 0.2 mm). Growth and development stage was defined by the appearance of a volcano-like structure followed by the protrusion of two appendices (2.0 mm until 7.0 mm of height). Maturation stage was defined by the gall maximum size, and acquisition of the horn-shape (9.5 ± 2.1 mm). Senescent stage was characterized by the brown color and presence of an escape channel (9.0 ± 1.2 mm, open galls). Samples of galls were fixed in FAA (Johansen, 1940), dehydrated in *n*-butyl series, and embedded in Paraplast^®^ (Kraus and Arduin, 1997). The samples were cross-sectioned (8–12 μm) and stained with astra blue and safranin solution (8:2, v/v) (Bukatsch, 1972, modified to 0.5%).

For scanning electron microscopy (SEM), samples of galls in all developmental stages were fixed in 4% Karnovsky (1965) (modified to phosphate buffer), post fixed in 1% osmium tetroxide, gradually dehydrated in ethanol series, CO_2_ critical point dried, and covered with 35 nm of gold (O’Brien and McCully, 1981). The samples were analyzed using the scanning electron microscope Jeol – JEM 6060.

### Histochemical Analysis

Fresh samples of horn-shaped galls at the late growth and development stage (the most metabolically demanding stage) were submitted to histochemical tests. The presence of IAA, indol-acetaldehyde (IAld), flavonoid derivatives and phenolics was tested using free-hand sections (room temperature). For IAA and IAld detection, the sections were treated with Ehrlich’s reagent (Leopold and Plummer, 1961) and *p*-dimethylaminocinnamaldehyde – DMAC (Schneider et al., 1972), respectively, for 5 min. IAA are stained in pink and IAld are stained in green, according to assays using commercial standards applied to thin layer chromatography (Bedetti et al., 2014). Flavonoid derivatives were detected by deep blue stain in sections incubated in 0.5% caffeine, 0.5% sodium benzoate, and 90% butanol for 5 min, and post incubated in 1% *p-*dimethylaminocinnamaldehyde (DMACA) in water, hydrochloric acid and ethanol (5:1:5) for 2 h (Feucht et al., 1986). Phenolics were detected in samples fixed in 2% ferric chloride in 95% ethanol (Gahan, 1984).

### Photosynthetic Yield by Chlorophyll Fluorescence Measurements

Fluorescence quenching analysis was performed in non-galled leaflets and galls (*n* = 5) at mature phase (at 8:00 am) using a modulated fluorescence imaging apparatus, Handy FluorCam PSI (Photo Systems Instrument, Czechia). The fluorescence parameters were measured on the surface of whole galls (not sectioned), and in hemisectioned galls (to measure photosynthetic yield in the innermost cell layers). Both whole and hemisectioned galls were analyzed to show the gradient of photosynthetic activity along the outermost cell layers to the inner cortex, nearest to the larval chamber. The maintenance of electron chains integrity, and the photosynthetic capacity of non-galled and galled tissues were demonstrated after adaptation to dark (30 min), and exposition to various light treatments following the software protocol – Quenching (Photo Systems Instruments, Version 2 ^1^). The following parameters were used in this study based on Genty et al. (1989), Lichtenthaler and Miehé (1997), and Oxborough (2004): *F*v/*F*m (maximum PSII quantum yield in dark-adapted state, where *F*v = *F*m-*F*_0_), (*F*′m-*F*′)/*F*′m (PSII operating efficiency, where *F*′m is the fluorescence signal when all PSII centers are closed in the light-adapted state and *F*′ is the measurement of the light-adapted fluorescence signal), Rfd (instantaneous fluorescence decline ratio in light, an empiric parameter used to assess tissue vitality), and NPQ_Lss_ (steady-state non-photochemical quenching in light).

## Results

### Gall Developmental Stages and Morphology

**FIGURE 1:**
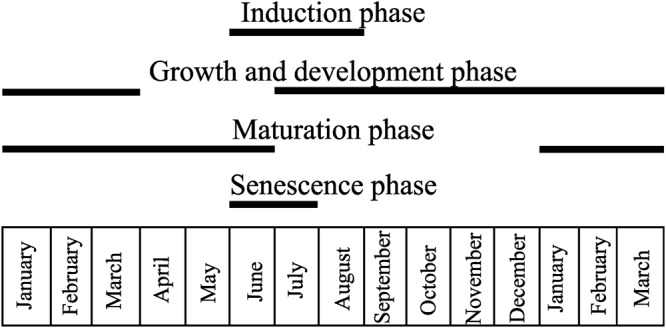
Univoltine life cycle of the horn-shaped gall induced by Diptera: Cecidomyiidae on the leaflets of *Copaifera langsdorffii*. Gall induction occurs from June to August, while the growth and development stage occurs from July to March, and overlaps with the beginning of the maturation stage, which lasts from January to June. The senescence stage occurs on June and July.

The galling Diptera: Cecidomyiidae induces a horn-shaped gall on the leaflets of *C. langsdorffii*, and has a univoltine life cycle along a year-time (**Figure 1**). Gall induction occurs from June to August concomitant to leaf flushing. The growth and development stage occurs from July to March and overlaps with the beginning of the maturation stage, which lasts from January to June. The senescent stage occurs during the heyday of the dry season in June and July.

**FIGURE 2:**
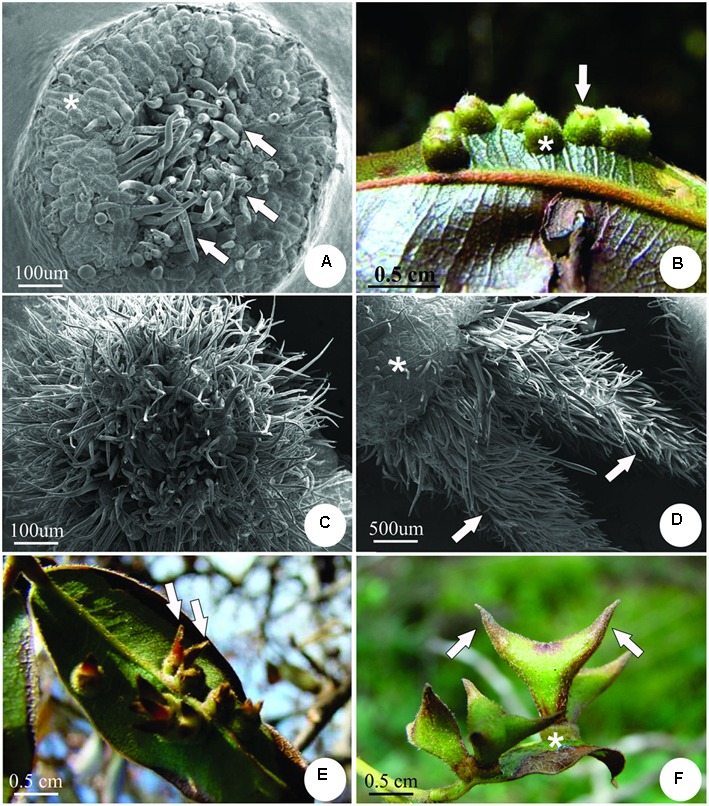
Developmental stages of the horn-shaped gall induced by Diptera: Cecidomyiidae on leaflets of *C. langsdorffii*. **(A)** Induction stage of the gall determined by the swelling of the leaflet lamina (asterisk), and redifferentiation of trichomes at the center. **(B)** Macroscopic aspect of the galls at the early growth and developmental stage, with the formation of the volcano-like structure (asterisk), and neoformed trichomes at the top (arrow). **(C)** Scanning electron micrograph showing detail of the volcano-like structure covered by neoformed trichomes. **(D)** Scanning electron micrograph showing detail of two appendices with a dense indumentum (arrows) emerging from the volcano-like structure (asterisk). **(E)** Macroscopic aspect of the galls at the growth and developmental stage with two appendices (arrows). **(F)** Mature galls are green, with two “horns” derived from the appendices (arrows), and the volcano-like structure remains at the base, forming a peduncle (asterisk).

The stage of gall induction is morphologically defined by the swelling of the leaflet lamina, with redifferentiated trichomes at the center (**Figure 2A**). Gall growth and development involve intense cell division and elongation to form a volcano-like structure covered by neoformed trichomes (**Figures 2B,C**). From inside the volcano-like structure, two appendices covered with a dense indumentum of non-glandular trichomes develop (**Figures 2D,E**). At maturation, galls reach their maximum size and the appendices turn into two lateral horn-like structures (**Figure 2F**). Galls may be isolated or clustered, occurring on both surfaces of the leaflets; they are pedunculated, closed, varying from light green to brownish-red. Each gall has a single larval chamber, which hosts one galling insect.

### Developmental Anatomy of the Galls

**FIGURE 3:**
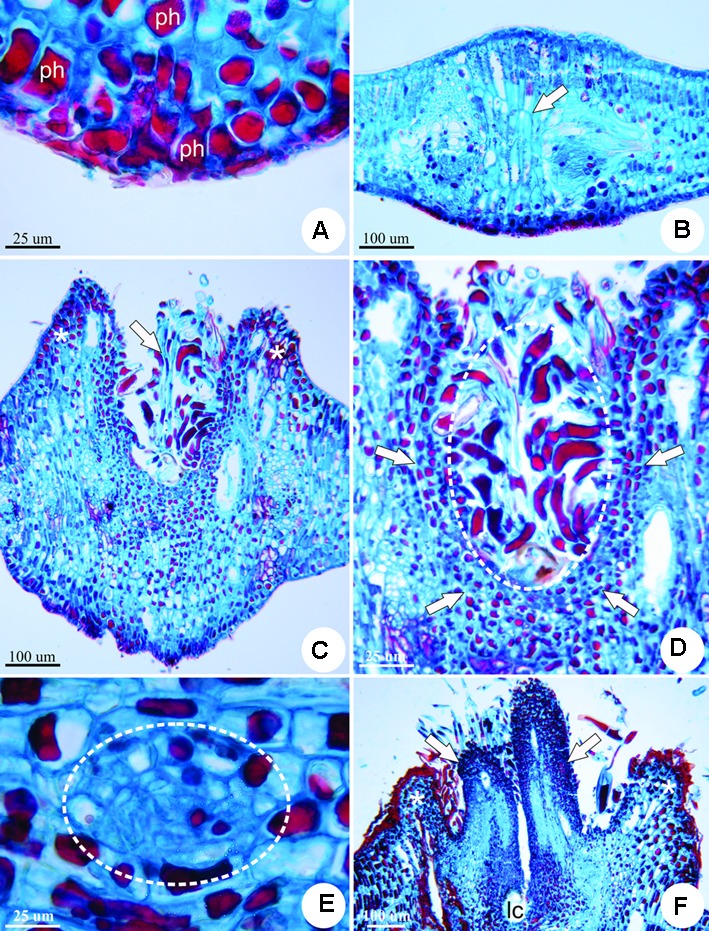
Anatomy of the horn-shaped gall induced by Diptera: Cecidomyiidae on the leaflets of *C. langsdorffii* in transverse sections. **(A)** Detail of phenolic substances (ph) accumulated at the site of induction. **(B)** Gall induction site defined by the swelling of the leaflet lamina, hypertrophy and elongation of mesophyll cells (arrow) and the accumulation of phenolics. **(C)** Early growth and developmental stage, with intense cell division and hypertrophy during the formation of a volcano-like structure (asterisks), and neoformation of trichomes (arrow). **(D)** Detail of small cells (arrows) nearest the larval chamber covered by trichomes (dotted line). **(E)** Detail of neoformed phloem bundle (dotted line) redifferentiated from the cortical parenchyma cells next to the larval chamber. **(F)** Redifferentiation of two appendices laterally to the larval chamber (lc) (arrows) that emerge from inside the volcano-like structure (asterisk).

The feeding activity of the galling larva stimulates the first detectable symptom of the induction phase of the horn-shaped gall, cell hypertrophy (**Figure 3A**) In addition, the hypertrophied cells have unidentified vacuolar substances (**Figure 3B**).

**FIGURE 4:**
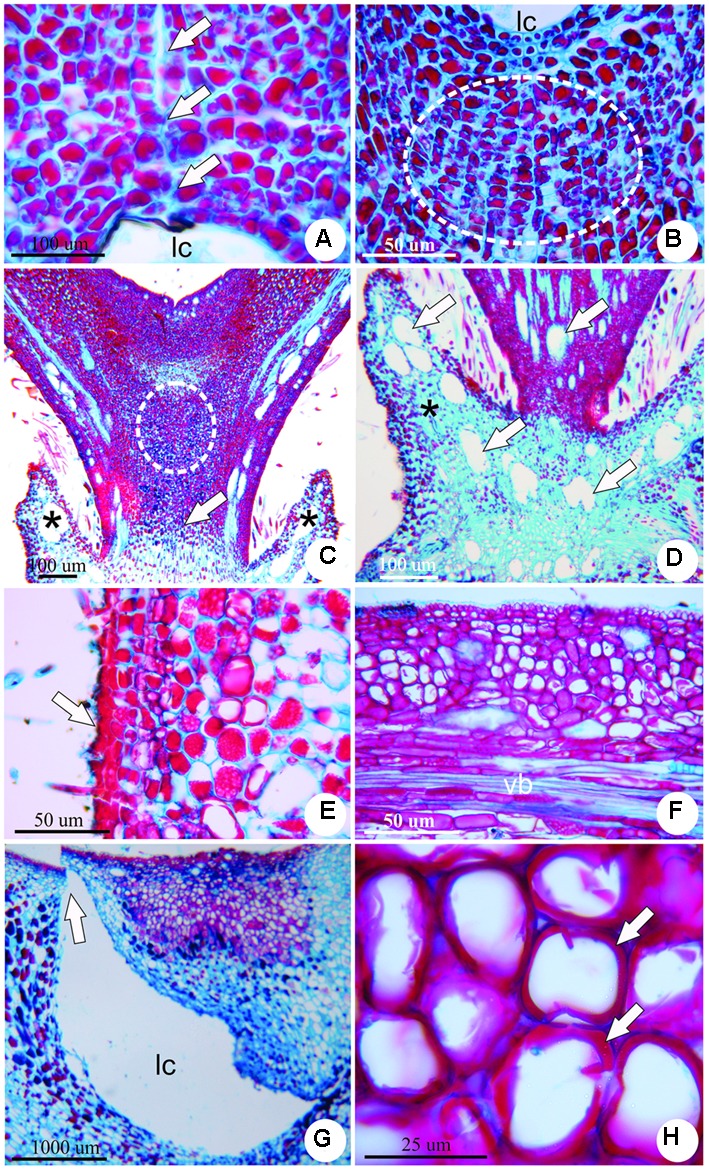
Anatomy of the horn-shaped gall induced by Diptera: Cecidomyiidae on the leaflets of *C. langsdorffii.* **(A)** Epidermal cells of the appendages fuse on the adaxial portion to close the larval chamber (arrows). **(B)** Meristem-like group of cells redifferentiate from parenchyma cells below the larval chamber (dotted line). **(C)** Gall at late growth and development stage, evidencing increment in cell divisions and elongation due to the activity of the meristem-like group of cells (dotted line), that protrude the gall body from inside the volcano-like structure (asterisks), forming a peduncle (arrow). **(D)** Detail of the volcano-like structure with numerous secretory cavities (arrows). **(E)** Detail of suberized tissue spot (arrow) on the outer surface of the mature gall. **(F)** Vascular bundle (vb) in the outer cortex of a mature gall. **(G)** Detail of the escape channel (arrow) built at the side of the larval chamber (lc) by the end of the maturation stage. **(H)** Detail of lignified cell walls (arrows) of nutritive cells in senescent gall.

The stage of growth and development occurs along 9 months and is featured by several important morphological events toward the establishment of the final shape of the gall. At the early phase of the growth and development stage, there is an increased periclinal division of mesophyll cells adjacent to the induction site. This hyperplasia, together with cell hypertrophy is responsible for the development of a volcano-like structure with neoformed trichomes (**Figure 3C**). These trichomes shelter the galling insect during this phase, for the larval chamber is yet to be completely closed. The cells nearest to the larval chamber are smaller than the cells of the outer layers of the volcano-like structure (**Figure 3D**). Neo-formed vascular bundles redifferentiate from parenchymatic cells in the cortex around the larval chamber (**Figure 3E**). Two groups of meristem-like cells redifferentiate inside the volcano-like structure and originate two appendices laterally to the larval chamber (**Figure 3F**). Epidermal cells accompany the growth of the appendices, and fuse on the adaxial portion of the structure closing a small larval chamber (**Figure 4A**). Below the larval chamber, a third meristem-like group of cells redifferentiates (**Figure 4B**) from parenchymatic cells, divide and elongate, protruding gall tissues upward from inside the volcano-like structure, and forming a peduncle (**Figure 4C**). Thus, the divisions of the meristem-like group of cells pushes the larval chamber up, together with the two lateral appendages in a continuum to determine the horn-shaped structure of the gall. The volcano-like structure remains at the base of the gall, where numerous secretory cavities occur (**Figure 4D**). Similar secretory cavities can also be observed at the outer cortex of the gall.

During the stage of maturation, gall size reaches its maximum size (9.5 ± 2.1 mm), and the horn-shaped structure is defined. Mature galls are green (sometimes with red or brown pigmentation), covered by simple epidermis, with sparse patches of periderm (**Figure 4E**). Gall cortex is eminently parenchymatic, with inconspicuous intercellular spaces, and a single larval chamber with one galling insect. Vascular bundles are distributed at the outer cortex of the gall (**Figure 4F**). Nutritive cells redifferentiate from the dermal system around the larval chamber.

By the end of maturation stage, galls enter the stage of senescence, which is characterized by the presence of an escape channel built from the expansion of the larval chamber toward the apex of the gall (**Figure 4G**). When the galling insect emerges through the escape channel, the walls of the nutritive cells around the larval chamber lignify (**Figure 4H**), and a suberized tissue totally replaces the epidermis.

### Histochemical Analysis

**FIGURE 5:**
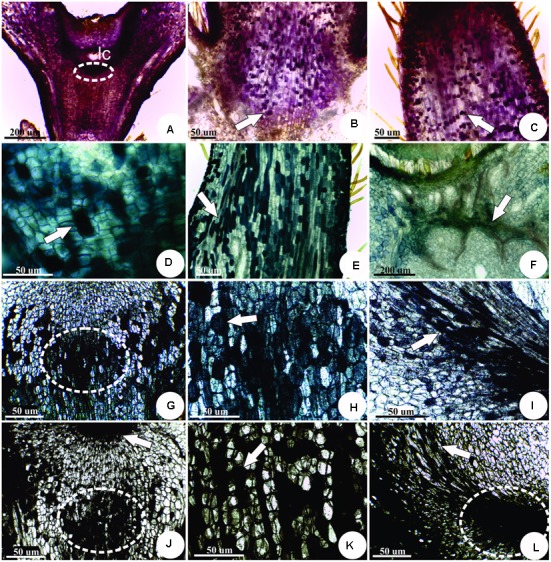
Histochemistry of mature horn-shaped gall induced by Diptera: Cecidomyiidae on the leaflets of *C. langsdorffii*. **(A–C)** sites of IAA accumulation detected by the pink staining with Ehrlich reagent. **(A)** General view, showing intense staining around the larval chamber (lc), at the meristem-like group of cells (dotted line). **(B)** Detail of reaction in the cells of the gall peduncle (arrow). **(C)** Detail of reaction in the cells of the gall “horn” (arrow). **(D–F)** Indol-acetaldehyde (IAld) accumulation evidenced in green by the staining with DMAC. **(D)** Detail of the reaction in the meristem-like group of cells (arrow). **(E)** Detail of reaction in the cells of the gall “horn” (arrow). **(F)** Detail of reaction in the vascular cells at the base of gall peduncle (arrow). **(G–I)** Flavonoids detected in dark blue by DMACA. **(G)** Detail of the reaction in the meristem-like group of cells (dotted line). **(H)** Detail of reaction in the cells of the gall peduncle (arrow). **(I)** Detail of the reaction in the flanks of the meristem-like group of cells (arrow). **(J–L)** Phenolics detected in black by ferric chloride reaction. **(J)** Detail of the reaction in the meristem-like group of cells (dotted line). **(K)** Detail of reaction in the cells of the gall peduncle (arrow). **(L)** Detail of the reaction in the flanks (arrow) of the meristem-like group of cells (dotted line).

The sites of IAA accumulation were detected by the pink staining with Ehrlich reagent, specially concentrated in the vascular bundles at the base of the larval chamber (**Figure 5A**), in the meristem-like cells (**Figure 5B**), and in the appendices (**Figure 5C**). The sites of IAld accumulation are evidenced by the green stain with DMAC in the meristem-like cells (**Figure 5D**), appendices (**Figure 5E**), and vascular bundles next to the larval chamber (**Figure 5F**). Flavonoid derivatives (**Figures 5G–I**) and phenolics (**Figures 5J–L**) detected by DMACA and 2% ferric chloride in 95% ethanol, respectively, overlapped at the sites of IAld and IAA accumulation.

### Chlorophyll Fluorescence and the Maintenance of Photosynthetic Activity in Gall Tissues

**FIGURE 6:**
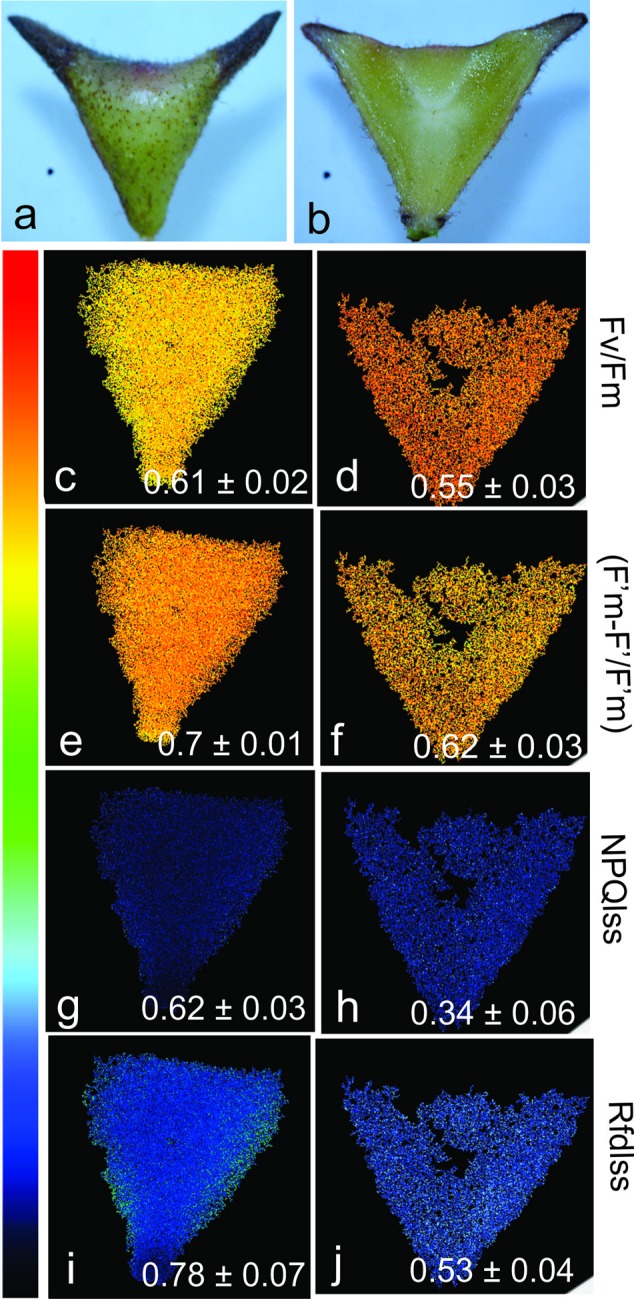
Chlorophyll fluorescence and photosynthetic activity in the horn-shaped gall induced by Diptera: Cecidomyiidae on *Copaifera langsdorffii*. **(a,b)** Whole gall and hemisectioned gall. **(c,d)** Fv/Fm (maximum PSII quantum yield in dark-adapted state) in outer and inner gall cortices. **(e,f)** (*F*′m–*F*′)/*F*′m (PSII operating efficiency) in outer and inner gall cortices. **(g,h)** Rfd (instantaneous fluorescence decline ratio in light, an empiric parameter used to assess tissue vitality) in outer and inner gall cortices. **(i,j)** NPQ_Lss_ (steady-state non-photochemical quenching in light) in outer and inner gall cortices.

The mature horn-shaped galls on *C. langsdorffii* are green (**Figures 6a,b**) and have potential to photosynthesize due to the presence of chlorophylls in most of the cortical cell layers. The values of *F*v/*F*m (0.61 ± 0.02 and 0.55 ± 0.03; whole and sectioned galls, respectively) (**Figures 6c,d**), as well as values of (*F*′m–*F*′)/*F*′m (0.7 ± 0.01 and 0.62 ± 0.03; whole and sectioned galls, respectively) (**Figures 6e,f**), indicate that the chlorophyllous tissue is able to photosynthesize throughout the gall cortex, except for the larval chamber and nearby cells. The steady-state non-photochemical quenching in light (NPQ_Lss_) (**Figures 6g,h**) is high in the whole galls when compared to the sectioned ones (0.62 ± 0.03 and 0.34 ± 0.06, respectively). The instantaneous fluorescence ratio decline in light (Rfd) (**Figures 6i,j**) indicates the decrease of tissue stability toward larval chamber in galls (0.78 ± 0.07 and 0.53 ± 0.04; whole and sectioned galls, respectively).

## Discussion

### Changes in Gall Morphology along the Univoltine Life Cycle

Galling insects are highly sensitive to phenological and physiological changes on their hosts, which, in the case of *C. langsdorffii*, lead to the establishment of seasonal syndromes for the occurrence of different gall morphotypes (Oliveira et al., 2013). The horn-shaped gall is the most abundant gall morphotype on *C. langsdorffii*, and it is induced exclusively on young leaves, whose tissues are responsive (*sensu* Weis et al., 1988) to the galling Cecidomyiidae. Thus, the period of leaf flushing of *C. langsdorffii* during the dry season is a true window of opportunity (*sensu* Mendonça, 2001) for the development of this gall morphotype.

Gall morphology has been the focus of studies in the Neotropics, and is an important tool for assessing and understanding the diversity of host plant-gall systems. The morphology of the horn-shaped galls is unique, and is particularly interesting for this gall shifts shape along the development. Such morphogenetical feature of shape-shifting has also been reported for the cup-shaped galls on *C. langsdorffii*, which are globoid during the early developmental stages, and turns to cup-shaped during maturity (Isaias et al., 2013). Nevertheless, differently from the horn-shaped gall, the cup-shaped gall has a very fast life cycle, and the shape-shifting occurs along 1-day time. Herein, we observe changes on the external morphology of galls to mark the transition from the induction stage toward the stages of growth and development, and maturity (Rohfritsch and Shorthouse, 1982; *sensu* Meyer and Maresquelle, 1983). Such marked shifts in shape, especially during growth and development phase, must involve deep anatomical reprogramming, which occurs in galls via cell redifferentiation toward new cell fates.

### Back to Meristematic Conditions and toward New Cell Fates

Classically, gall shape has been understood as a response of plant cells to the feeding behavior of the gall inducer, which stimulates plant tissues to grow toward a new structure (Rohfritsch and Anthony, 1992). However, the high diversity of gall shapes found in nature indicates that such classic concept does not always explain the diversity of gall morphotypes found in nature. Cell hypertrophy and tissue hyperplasia are the most common plant responses to the stimuli of galling herbivores (Mani, 1964; Rohfritsch, 1992; Isaias et al., 2014b; Oliveira et al., 2016), and were also observed during the development of the horn-shaped galls on *C. langsdorffii*. The protrusion of the galls from the leaf lamina requires a set of periclinal cell divisions and anticlinal cell elongation, as observed herein, and in other Neotropical galls (*cf*. Isaias et al., 2014b). Such cell divisions are observed to occur especially due to the activity of meristem-like group of cells, which remain clustered below the larval chamber, while the cells next to it undergo differentiation. As formative plant tissues, meristems are composed by cells capable of dividing and giving rise to new cells (Scofield and Murray, 2006), with defined patterning and genetic regulation (Miwa et al., 2008), features of the meristem-like group of cells in the horn-shaped galls.

Each gall morphotype has specific patterns of cell division and elongation during the transition of the stage of induction toward the stage of maturation, when the final shape of the gall is defined. The intense activity of the meristem-like groups of cells determines the shape of the horn-shaped galls. Meristematic cells were previously reported in galls on *Guapira opposita*, forming multiple buds on the surface of the gall, and producing numerous leafy projections, which determine the rosette gall morphotype (Fleury et al., 2015). However, the developmental sites of the galls on *G*. *opposita* are the axillary buds, where meristem potentialities are naturally expressed. The horn-shaped galls are induced on leaflets, where meristematic tissues are lacking, and the presence of the meristem-like groups of cells in gall structure indicates cell redifferentiation (*sensu* Lev-Yadun, 2003) during gall development. The perspective of the reacquisition of the meristematic potentiality involves specific genetic control as proved for the establishment and maintenance of the apical meristem (Raghavan, 1986; Fosket, 1994). Also, the development of the two horn-like appendages resembles the general development of leaf primordia (Moon and Hake, 2011), with differences on the final structure.

The redifferentiation of tissues in gall developmental sites involves a cascade of events triggered by reactive oxygen species (ROS) and followed by the accumulation of phenolic derivatives. The enhanced levels of ROS impair the homeostasis of gall tissues, which may trigger the accumulation of ROS scavengers, such as the phenolics (Isaias et al., 2015). The accumulation of phenolics ends up inhibiting the activity of IAA-oxidases (Hori, 1992), thus increasing the cellular levels of auxins and their precursors (Isaias et al., 2015). In the leaflet galls on *Piptadenia gonoacantha*, sites of intense cell division and hyperplasia accumulate phenolics, IAA and its precursor, thus adding a topological evidence of the role of phenolics on the control of auxin levels during gall development (Bedetti et al., 2014). Similarly, the histochemical profiles of the horn-shaped galls on *C. langsdorffii* show that phenolics, IAA and IAld accumulate in the meristem-like group of cells below the larval chamber. Such group of cells can be recognized and its ectopic occurrence and abnormal activity originate the horn-shaped gall, a new plant organ with singular phenotype. Even though the host leaflet anatomy deeply changes toward this gall morphotype, some cell lineages express conservative functions, as the photosynthetic activity, which helps maintaining the stability of gall metabolism.

### Conservative Functionalities beyond the Unusual Structure

Alterations on the phenotype of the leaflet lamina toward the horn-shaped gall require dramatic changes on the structure and functions of *C. langsdorffii* cells and tissues. Leaves are organs designed to effectively exchange gasses with the surrounding environment, and to photosynthesize at high levels, thus functioning as a carbon source for plants. Conversely, galls are structures that protect and nurture the galling herbivores, often seen as carbon sinks of their host organs due to photosynthetic deficiency (Carneiro et al., 2014), or by the high demand of energy to sustain a vigorous growth and metabolism. Previous studies with the horn-shaped galls indicate that the photosynthetic activity occurs at low levels in gall tissues when compared to non-galled leaflets, despite the deep structural changes (Castro et al., 2012).

The reduction of the *F*v/*F*m and (*F*′m–*F*′)/*F*′m values in the sectioned horn-shaped galls indicates a decrease of photosynthetic potential from gall periphery toward the inner cortex. Additionally, the low intensity of light that reaches the innermost layers of the horn-shaped galls, and the underdeveloped photosynthetic apparatus of the meristem-like groups of cells should explain the low values of *F*v/*F*m, (*F*′m–*F*′)/*F*′m and Rfd, as proposed by Haitz and Lichtenthaler (1988) for other stress-generating factors. The photosynthesis in green galls provides oxygen to avoid hypoxia and consume carbon dioxide to avoid hypercarbia in their tissues, thus working as a metabolic strategy to maintain the stability of the new organ (Pincebourde and Casas, 2016; Oliveira et al., 2017). Even though the horn-shaped gall is large-sized, with highly compacted tissues, it seems to have a functional metabolism of carbon dioxide and oxygen. Such efficiency explains how abundant and large galls withstand their long life cycles on *C. langsdorffii* as their photosynthesis rates should help reducing intra-plant and inter-gall competition, as hypothesized for *A. longifolia* galls (Haiden et al., 2012).

## Conclusion

The biotic stress induced by the galling Cecidomyiidae on the structure and physiology of the leaflets of *C. langsdorffii* lead to distinct functionalities of plant cells and tissues, culminating in a new organogenetic pattern. During the univoltine life cycle of the galling Cecidomyiidae, we observe drastic changes on the external morphology of the galls with a marked transition from the induction stage toward the stages of growth and development, and maturity. Such marked shifts in shape require cell dedifferentiation and redifferentiation at the center of the gall, especially during growth and development phase, when the gall body emerges from inside the volcano-like structure. Subsequently, new meristematic centers at the flanks originate the parenchymatic horns. These meristematic zones drive deep anatomical reprogramming toward the reacquisition of the meristematic condition. The conservative functionalities of the host organ in galls, as the photosynthesis activity, maintain tissue homeostasis by avoiding hypoxia and hipercarbia in the highly compacted gall tissues. New frontiers on the functioning of ectopic meristematic sites, such as the meristem-like group of cells in the horn-shaped galls on *C. langsdorffii* should rely on the investigation of molecular mechanisms of cell signaling for the determination of the unusual structural patterns of complex galls.

## Author Contributions

RC, RI, AM, and DO designed the sample. RC, AM, and DO field sampling. RC, RI, AM, and DO data analysis. RC, RI, AM, and DO Wrote the manuscript.

## Conflict of Interest Statement

The authors declare that the research was conducted in the absence of any commercial or financial relationships that could be construed as a potential conflict of interest.

## Footnotes

1 www.psi.cz
